# Occult breast cancer presenting as axillary lymphadenopathy – Case Report

**DOI:** 10.1016/j.ijscr.2022.107677

**Published:** 2022-09-19

**Authors:** Rita Camarneiro, Ágata Ferreira, Manuel Barros, Margarida Brito e Melo

**Affiliations:** a Surgery Department, Unidade de Caldas da Rainha - Centro Hospitalar do Oeste, Rua Diário de Notícias 2500-176, Caldas da Rainha, Portugal; b Oncology Department, Unidade de Caldas da Rainha - Centro Hospitalar do Oeste, Rua Diário de Notícias 2500-176, Caldas da Rainha, Portugal

**Keywords:** Occult breast cancer, Axillary lymph nodes, Breast, Female, Breast MRI

## Abstract

**Introduction:**

Occult breast carcinoma (OBC) is a rare entity and therefore generates discussion regarding diagnosis, approach, and prognosis. This article aims to present a case of OBC and reviews some concepts discussed in the literature.

**Presentation of case:**

43-year-old woman with right axillary adenopathies, without further complaints, whose biopsy shows a lymph node metastasis from invasive ductal carcinoma of the breast. Breast study, breast RMI and FDG-PET did not identify the primary tumour. As decided by a multidisciplinary team, the patient underwent neoadjuvant chemotherapy, axillary surgery, breast radiotherapy and hormone therapy. Four years after surgery, the patient has no evidence of the primary tumour and no axillary recurrence.

**Discussion:**

OBC was described in 1907. Although the best therapeutic approach is widely discussed in the literature, it is consensual that as long as the existence of a primary tumour is excluded by breast MRI, the conservative approach (excision of axillary adenopathy and breast and axillary radiotherapy) is more advocated.

**Conclusion:**

Breast cancer must be considered in the differential diagnosis of a patient with axillary lymphadenopathy. The conservative approach of OBC is the preferred since breast MRI does not identify any suspicious lumps.

## Highlights

• Occult breast carcinoma is rare.
• Breast carcinoma can manifest through axillary adenopathy.
• Conservative approach is the preferred.

## 1 Introduction

Occult breast carcinoma (OBC) is defined as carcinoma of unknown primary, consistent with metastatic carcinoma of breast origin without clinical or imaging evidence of a definitive breast primary tumour. It is extremely rare, less than 1 %, and it presents primarily as a metastatic axillary lymph node [1], [2], [3], [4], [5], [6].

The diagnosis, treatment and prognosis of OBC still remain unclear. There have been studies and meta-analyses comparing the most invasive approach, which include mastectomy and axillary lymph node dissection versus breast-conserving surgery and radiotherapy. The latter does not seem to be inferior [2], [3], [5], [6].

Since it is an infrequent entity, in which the therapeutic approach is still controversial, the main objective of this work is to disclose a case of occult breast tumuor whose first manifestation was an axillary adenopathy and thus increase the evidence on options for treating these cases.

This case is reported as follows SCARE Guidelines [7].

## 2 Presentation of case

43-year-old woman, without past medical history, namely, gynaecological or oncology history. Family history with a second-degree relative diagnosed with breast cancer (the age at diagnosis is unknown).

The patient was referred to a breast surgery consultation due to painless, hard-right axillary mass with one month of evolution.

There were no other symptoms or signs associated, namely, other adenopathies.

**Fig. 1:**
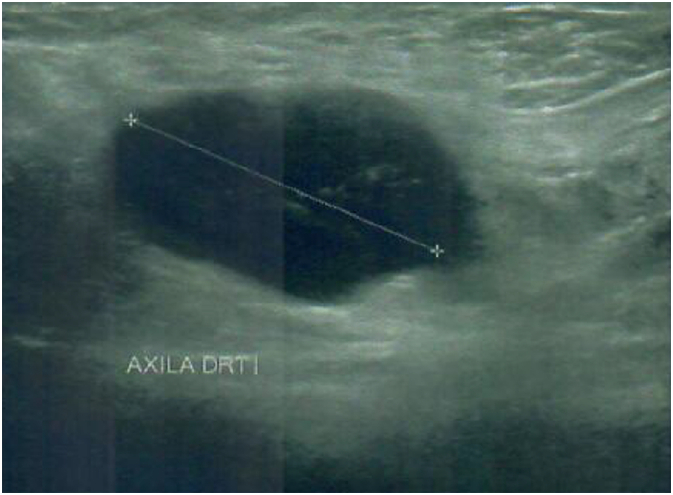
Right axillar lymph node, 20.3X30.7mm.

**Fig. 2:**
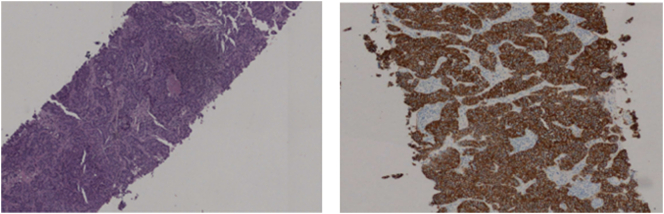
Histologic specimens of fine needle aspirating biopsy of right axillary adenopathy – metastasis of invasive ductal breast carcinoma, positive estrogen and progesterone in 100 % of tumour cells, c-erb B2 3+, Ki67 in 80 % of tumour nuclei.

The diagnostic procedure was initiated with a laboratory investigation that showed no pathological findings. Axillary ultrasound showed a right axillary adenopathy conglomerate, measuring 20.3X30.7 mm, with suspicious ultrasound characteristics (Fig. 1). Fine needle biopsy of the axillary mass was performed, and the histological analysis was compatible with metastatic invasive ductal breast carcinoma (Fig. 2). Immunohistochemical profile with 100 % positivity for estrogen and progesterone receptors, c-erb B2 3+ and Ki-67 80 % (Luminal B like). Breast ultrasound, mammography and MRI was performed showing no pathological changes. PET-CT, in addition to abnormal uptake in the axillary lesion, showed no pathological FDG uptake in the breast parenchyma.

Given these findings and according to the 8th edition of the American Joint Committee on Cancer (AJCC) – TNM staging system for breast cancer, we are faced with an occult breast tumour (cT0), cN2 (fixed ipsilateral axillary adenopathy), M0 (no distant metastases), which corresponds to a stage IIIA. The patient was evaluated by a multidisciplinary board, and it was decided to start neoadjuvant systemic chemotherapy - four cycles of neoadjuvant chemotherapy according to the EC-P dose-dense protocol (Epirrubicin, Cyclophosphamide and Paclitaxel) and Trastuzumab (it kept for one year) - and subsequent axillary lymphadenectomy followed by radiotherapy and hormone therapy. Although the recommendations also suggest performing a mastectomy in this case, after discussion between the multidisciplinary team and the patient, the patient chose not to undergo a mastectomy.

**Fig. 3:**
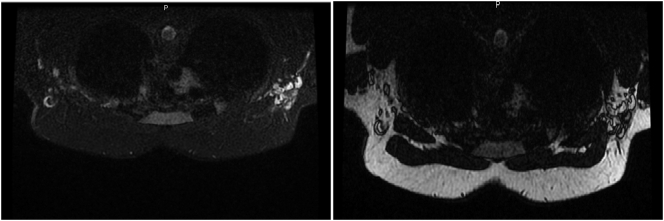
Breast MRI after neoadjuvant chemotherapy, maintaining right axillary adenopathic conglomerate, without breast lesions.

After systemic chemotherapy, breast MRI was repeated, which showed a decrease in the right axillary adenopathy conglomerate, however with no findings of breast lesions (Fig. 3).

Uneventful right axillary lymphadenectomy was performed. In the surgical specimen, 19 lymph nodes were isolated, without tumour.

Three weeks after the surgery, since the patient preferred to preserve the breast, she started radiotherapy directed to the right axilla and breast (50 Gy/ 25 fractions). Hormone therapy with Examestan (25mg/day) and Trastuzumab was maintained.

Four years after therapy, the patient remains under follow-up with ultrasound, mammography and breast MRI, showing no signs of lymph node recurrence neither pathological signs of primary breast tumour.

## 3 Discussion

Axillary adenopathies aetiology can be challenging, given the variety of underlying benign and malignant etiologies [7]. Among the malignant causes of axillary adenopathies, stand out the following: breast cancer, thyroid, lung, pancreas, stomach, colon, uterus, ovaries [4].

In a women with isolated axillary adenopathies, breast cancer should be suspected and, a breast study (mammography and breast ultrasound) should be performed [3]. The sensitivity of the breast study to identify a neoplastic lesion is approximately 75%, being even lower in patients with denser breast [3]. Completing the study with breast MRI increases the detection sensitivity to close to 100% [3], [4], [8].

The biopsy of the suspicious lymph node, and the subsequent immunohistochemical study, will allow the diagnosis [2]. If the biopsy shows metastasis of a breast tumour, without the breast exams identifying the primary tumour, then we are dealing with an OBC. In these cases, an additional study with a thorax-abdomen-pelvic CT scan or FDG PET, may be conducted in an attempt to detect lesions that were not identified in the previous exams [4].

Some authors also argue that the exclusion of the existence of a primary tumour through breast MRI at the time of staging is essential [3], [4].

OBC was first described in 1907 by Halsted et al. Since then the diagnostic and therapeutic approach has generated discussion and its incidence has decreased with the development of diagnostic modalities [3], [4], [5], [6], [8].

The appearance of axillary lymph node metastases in the absence of a primary breast tumour is uncommon [5], [8]. The clinical case presented here is a true OBC, since after performing a breast study, breast MRI, thoraco-abdomino-pelvic CT scan, and FDG PET no suspicious breast lesion was identified.

According to the 8th edition of the American Joint Committee on Cancer (AJCC) it is staged as T0 N1-2 M0, which corresponds to stage II–III [9], [10].

The treatment should be based on the stage of the disease [4]. The case of the patient presented was categorized as stage III (cT0, cN2a, M0).

OBC management varied over time. Initially mastectomy was the treatment of choice. However, over the years, evidence has emerged that the conservative approach has superior results (overall survival and disease-free survival) [3], [4].

Radiotherapy is essential when breast conservative therapy is planned [3], [4]. There is no consensus on which area should be irradiated: since no lesion is identified in the breast, it is also discussed in the literature whether it should also be irradiated [4].

As in breast cancer, in OBC, specific therapies according to the molecular subtype help in the locoregional and systemic control of the disease [3], [4].

Here, according to lymph node staging, neoadjuvant therapy should be performed [10]. After undergoing neoadjuvant therapy, the patient was re-staged with breast MRI. Although smaller, the right axillary conglomerate was maintained. Right axillary surgery was performed with radical oncological lymphadenectomy since there was a conglomerate of nodes. After conservative approach to OBC, as defended by many authors, the patient was also submitted to breast and axilla radiotherapy. As it is a tumour with positive HER-2 receptors, the patient underwent target therapy with Trastuzumab and is still under endocrine therapy (Examestane).

Some authors consider that the prognosis of OBC depends on the lymph node staging (N) and the location of the metastatic nodes [3]. The involvement of supraclavicular and internal mammary ganglion chains is associated with a worse prognosis, and higher risk of local and distant recurrence after conservative therapy of OCB [3], [4], [8].

OBC, when limited to axillary metastases usually has a good prognosis after breast conservative therapy [3].

In most studies in which the initial breast assessment included MRI (as in the case presented here), the recurrence rate after conservative OBC therapy was around 9% [3].

Similar to other breast cancer types, the major prognostic factors in OBC are pathological and molecular types of primary cancer, number of axillary lymph nodes positives, involvement of the supraclavicular and internal mammary ganglion chains, and distant metastases [4].

The prognosis of OBC is comparable to stage II disease, with 5 years survival of 50‐–87 %, depending on the affected lymph nodes: localization and number of the metastatic lymph nodes [4].

## 4 Conclusion

Breast cancer must be considered in the differential diagnosis in a patient with a sole presentation of axillary lymphadenopathy.

Despite the evolution of complementary diagnostic tests, namely breast MRI, although rare, there are still cases of OBC.

The conservative approach (axillary lymph node dissection associated with breast RT) is currently the preferred approach when breast MRI does not identify the primary lesion.

## Consent

Written informed consent was obtained from the patient for publication of this case report and accompanying images.

## Ethical approval

The article is exempt from ethical approval and has the patient's consent for publication.

## Funding

The article has no sponsors.

## Author contribution

Rita Camarneiro, MD – article writing, assistant surgeon in the patient's surgery.

Ágata Ferreira, MD - assistant surgeon in the patient's surgery

Manuel Barros, MD. patient's oncologist.

Margarida Brito e Melo, MS – patient's surgeon, director of surgery department of Centro Hospitalar do Oeste – Caldas da Rainha.

## Guarantor

Not applicable.

## Research registration number

Not applicable.

## Declaration of competing interest

None of the authors have conflicts of interest.
